# AI-Assisted Identification of a Putative Allosteric Ligand Targeting the CDK4/Cyclin D1 Protein–Protein Interface

**DOI:** 10.3390/ph19060970

**Published:** 2026-06-22

**Authors:** Barış Kurt

**Affiliations:** Department of Chemistry, Mus Alparslan University, 49250 Muş, Türkiye; b.kurt@alparslan.edu.tr

**Keywords:** CDK4/Cyclin D1, protein–protein interaction, allosteric drug discovery, RapidFunnel-AI, Q-Fold Thermo-Core, contact retention, molecular dynamics, MM-GBSA

## Abstract

**Background/Objectives**: First-generation CDK4/6 inhibitors (palbociclib, ribociclib, abemaciclib) target the conserved ATP-binding pocket of CDK4 and, despite clinical success, are limited by acquired resistance and insufficient exploration of alternative regulatory sites. This study aimed to identify a putative allosteric small-molecule candidate at the CDK4 αE-helix–Cyclin D1 α1-helix protein–protein interaction (PPI) interface within the CDK4/Cyclin D1/p21 ternary complex using RapidFunnel-AI, a decision-interpretable virtual-screening pipeline. **Methods**: Starting from 50,000 ChEMBL 33 molecules, the pipeline sequentially applied a Q-Fold/RapidFunnel topological Tanimoto scan based on clinical CDK4/6 inhibitor motifs, fragment-level electronic-property enrichment, ADMET/PAINS filtering, dry Vina-GPU docking, hydration-mediated AutoDock-GPU (Version 1.6) docking, explicit-solvent molecular dynamics, contact-retention analysis, and MM-GBSA energy decomposition. The Q-Fold Thermo-Core surrogate model provided fragment-level enrichment, predicting the HOMO–LUMO gap (R^2^ = 0.93) and isotropic polarizability (R^2^ = 0.98) on QM9. Candidate selection did not rely on the lowest docking or MM-GBSA score alone, but on pose persistence, contact continuity, and energy-component consistency. **Results**: The workflow reduced the initial library to 43 topologically prioritized candidates, 25 ADMET/PAINS-filtered ligands, and 9 docking-derived complexes for MD validation. Ligand_020 emerged as the only candidate that preserved a persistent binding mode at Site 2 during a 500 ns simulation—an interface engagement reproduced across three independent 500 ns replicates with no full dissociation in any replicate—with a protein Cα RMSD of 2.88 ± 0.32 Å, a ligand heavy-atom RMSD of 3.56 ± 0.28 Å, and a van der Waals-dominated MM-GBSA profile (ΔGbind = −28.23 ± 3.57 kcal/mol). In contrast, palbociclib and ribociclib, forcibly placed at Site 2 as negative controls, lost most initial contacts within 5 ns and tended to detach despite more favorable MM-GBSA values. **Conclusions**: These results suggest that single-score docking or MM-GBSA ranking can generate false positives at shallow PPI interfaces. By integrating AI-assisted prioritization, multipocket docking, explicit-solvent MD, contact-retention analysis, and energy-component consistency, RapidFunnel-AI nominated Ligand_020 as an experimentally testable putative allosteric hit targeting the CDK4/Cyclin D1 interface, offering a reusable platform for PPI-focused oncological drug discovery.

## 1. Introduction

Cyclin-dependent kinase 4 (CDK4) is a key regulator of the G1-to-S phase transition of the cell cycle and shows aberrant activity in several oncological pathologies, particularly breast cancer [1]. The clinical success of ATP-competitive inhibitors such as palbociclib, ribociclib, and abemaciclib has placed CDK4 at the center of targeted cancer therapy. However, all of these first-generation agents act on the evolutionarily conserved active ATP-binding pocket of the enzyme. Clinical data indicate that prolonged selective pressure on this region inevitably promotes secondary resistance mutations in the target protein, and that patients may gradually lose therapeutic response [2,3]. This situation highlights the urgent need for alternative molecules with new mechanisms of action that can move beyond the limitations of the ATP pocket in oncological drug development.

One of the most rational strategies for overcoming resistance mechanisms is to move beyond the conventional active site and target allosteric regions or protein–protein interaction (PPI) interfaces. CDK4 must form a complex with Cyclin D1 to perform its cellular function and reach its active state. Therefore, the CDK4/Cyclin D1 interface provides an attractive allosteric target that may modulate cellular signaling pathways with greater selectivity [4]. However, unlike classical enzyme pockets, PPI interfaces are broad, shallow, and flexible; designing specific small molecules for these regions using only static structure-based lock-and-key models is challenging and prone to high false-positive rates [5].

A major bottleneck in modern pharmaceutical drug discovery is that experimental screening of the vast chemical space is costly and time-consuming [6,7]. Artificial-intelligence- and machine-learning-based prioritization approaches have therefore become not merely accelerators but central components of decision-making, particularly in small-molecule hit discovery, scaffold hopping, and repositioning strategies. In this study, AI was not used as a secondary analytical layer applied after docking; rather, it functioned as a decision engine at the start of the screening campaign to reduce chemical space to a biologically meaningful subset. Local topological motifs learned from clinical CDK4/6 inhibitors, surrogate predictions representing quantum-chemical electronic tendencies, and drug-likeness filters were combined to reduce the high false-positive burden generated by conventional brute-force docking approaches [8,9].

Multistage, funnel-based, sequential in silico protocols are required to assess the biological plausibility of hit candidates obtained from AI-based prescreening. Molecular docking statically evaluates whether a ligand can fit into the target pocket, whereas ADMET (absorption, distribution, metabolism, excretion, and toxicity) filtering removes toxic or pharmacokinetically problematic molecules with limited clinical potential [10]. Molecular-dynamics (MD) simulations, the final and most critical step, mimic the motions of the target protein and ligand in a physiological environment surrounded by water and ions. By reducing the uncertainty associated with static docking, MD simulations constitute a gold-standard approach for testing the time-dependent thermodynamic stability of complexes and selecting the most robust candidates [11].

The primary aim of this study was to identify experimentally testable putative allosteric hit candidates targeting the CDK4/Cyclin D1 PPI interface through a pharmaceutically interpretable, reproducible, AI-assisted drug-discovery pipeline. A raw library of 50,000 molecules obtained from ChEMBL was first prioritized with Q-Fold/RapidFunnel-AI using an internal Tanimoto-based topological scan against clinical CDK4/6 inhibitors as references; this step yielded 43 candidates. These candidates were then passed through ADMET/PAINS filtering, multipocket docking evaluation, and explicit-solvent MD simulations. Thus, this study not only proposes a single ligand but also presents a pharmaceutically applicable decision architecture showing how AI and physics-based simulations can be integrated for targets such as PPI interfaces, where classical docking scores have limited reliability.

## 2. Results

### 2.1. Multistage Screening Output of the RapidFunnel-AI/Q-Fold Protocol

The raw pool of 50,000 molecules from ChEMBL 33 was progressively narrowed using the multilayer RapidFunnel-AI/Q-Fold prioritization protocol (Table 1). The internal Q-Fold/RapidFunnel topological scan (Tanimoto ≥ 0.30 using palbociclib, ribociclib, and abemaciclib as references) selected 43 candidates from 50,000 molecules. The ADMET filter (maximum one Lipinski violation, TPSA ≤ 140 Å^2^, rotatable bonds ≤ 10, PAINS = 0) was then applied to these 43 candidates, yielding 25 ligands that were submitted in parallel to both dry and hydration-mediated docking workflows.

Docking was performed at the four pocket centers defined by PrankWeb/P2Rank on the 6P8H ternary complex (CDK4/Cyclin D1/p21^Cip1) (Table 2). In total, 25 ligands were evaluated across four pockets and two docking protocols, and Gypsum-DL protomer/tautomer variants produced 336 independent docking runs. Four complexes from the dry workflow and five complexes from the hydration-mediated workflow were transferred to the MD stage. Systems showing early ligand drift during MD simulations were terminated within 20–50 ns, whereas Ligand 020 (dry docking + Site 2 combination) was the only candidate to complete the full 500 ns production simulation with preserved structural integrity.

### 2.2. MD-Based Rejection of Unstable Docking-Derived Candidates

During MD validation of the nine complexes selected from the ChEMBL library after docking, all candidates except Ligand 020 were eliminated because of structural instability (Table 3). Ligand 013 showed complete unbinding from the target pocket, with a progressive RMSD increase reaching 13.7 Å in the late phase of the simulation and preservation of the dominant Cyclin D1 Arg26/8 contact in only 46% of frames. Ligand 023 abandoned its docking pose within the first 150 frames, formed a surface-sliding plateau in the 7.5–8.5 Å range, and retained only shallow hydrophobic surface contacts (Cyclin D1 Arg26/8: 47%, CDK4 Ala130/352: 22%, Cyclin D1 Val27/9: 11%). Ligand 021 reached a ligand RMSD of 8.9 Å and an RMSF value as high as 8.08 Å. The remaining lower-ranked candidates displayed similar rapid unbinding and solvent-exposure patterns within the first 20–50 ns.

RMSD/RMSF time series, contact-analysis outputs, and MM-GBSA calculation summaries for all rejected candidates are openly available in the Supplementary Materials and in the GitHub repositories listed in Table 3. MM-GBSA values for the rejected candidates are not discussed in the main text because, in the absence of structural locking, negative binding energies arising from transient hydrophobic surface contacts cannot be interpreted as evidence of stable binding; these values were nevertheless deposited for transparency and reproducibility.

### 2.3. Structural Stability of the Ligand 020 Complex

**Figure 1:**
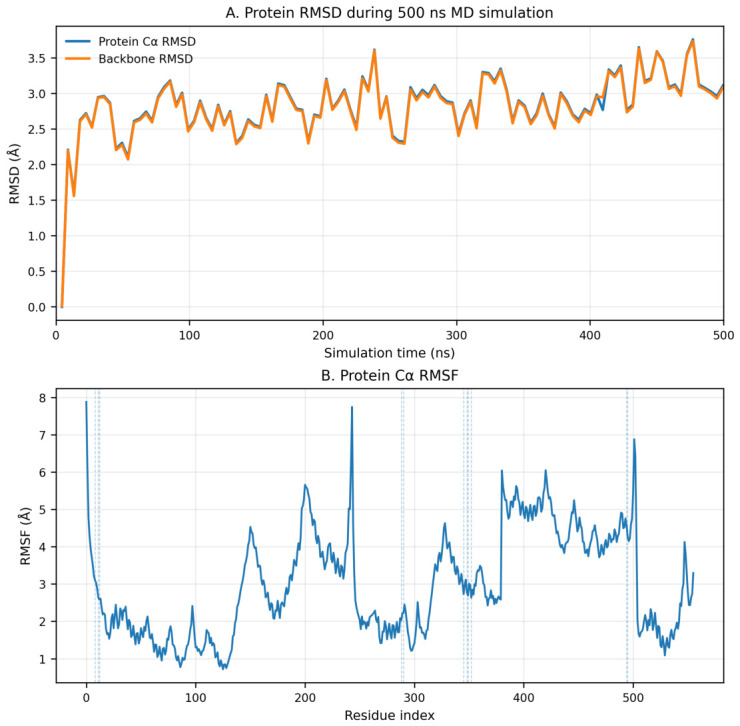
Structural stability of the CDK4/Cyclin D1/p21 complex during the 500 ns MD simulation of Ligand 020 at Site 2. (**A**) Protein Cα RMSD and backbone RMSD as a function of simulation time. (**B**) Protein Cα RMSF profile; dashed vertical lines indicate major ligand-contacting residue positions.

Ligand 020 remained in the Site 2 pocket throughout the 500 ns production simulation (Figure 1A). The Cα RMSF profile showed that residue-level mobility was mainly localized to flexible loop and terminal segments, while the protein core retained a compact architecture throughout the trajectory (Figure 1B). All contact residues on the binding surface, including Cyclin D1 Arg26, remained within low fluctuation bands of 2–3 Å. The high-RMSF regions of the system (the ~7.8 Å peak around residue 240 and the continuity break around residue 380) corresponded to flexible loops not modeled because of missing electron density in the crystal structure, and to interchain transition regions; these regions are located away from the ligand-binding pocket.

**Figure 2:**
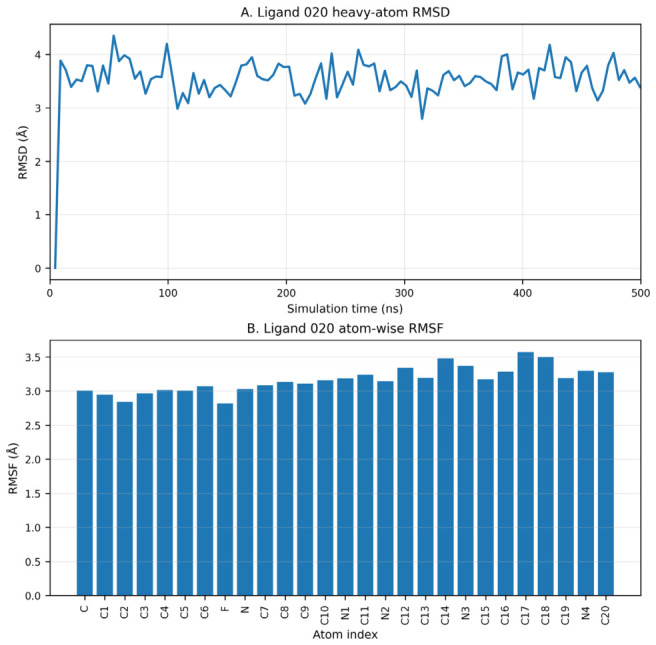
Dynamic stability of Ligand 020 in Site 2 during the 500 ns MD simulation. (**A**) Ligand heavy-atom RMSD after protein Cα alignment. (**B**) Atom-wise ligand RMSF, showing a homogeneous fluctuation profile without localized excessive flexibility.

Ligand 020’s heavy-atom RMSD was locked within a narrow band of 3.56 ± 0.28 Å (Figure 2A), and no progressive drift, regional jump, or solvent escape pattern was observed during the simulation. Ligand–atom RMSF values were distributed homogeneously across the 27 heavy atoms, ranging from 2.82 to 3.57 Å, with an average of 3.16 Å (Figure 2B). No localized excessive flexibility, fragmentation, or conformational collapse was observed.

### 2.4. Dynamic Contact Profile of Ligand 020 at Site 2

**Figure 3:**
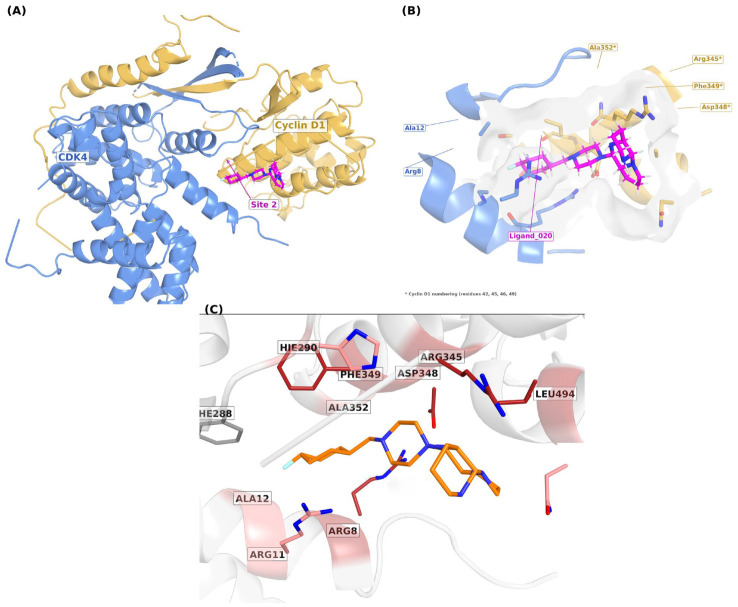
Structural context and binding mode of Ligand_020 at Site 2. (**A**) Global view of the CDK4/Cyclin D1 complex showing the location of Site 2 at the protein–protein interface. CDK4 is shown in blue, Cyclin D1 in gold, and Ligand_020 in magenta. (**B**) Close-up surface representation of the Site 2 pocket. Key interface residues are labeled using the canonical UniProt residue number/MD-composite residue number convention: Cyclin D1 Arg26/8 and Ala30/12, and CDK4 Arg123/345, Asp126/348, Phe127/349, and Ala130/352. (**C**) Final-frame binding mode of Ligand_020 after the 500 ns production MD simulation, showing representative ligand–protein heavy-atom contacts within 4 Å. Ligand_020 is shown as orange sticks. The snapshot corresponds to the final production frame (t = 500 ns).

The binding mode of Ligand 020 in the Site 2 pocket was visualized from two orientations using the final simulation frame (t = 500 ns) (Figure 3). The contact network annotated in PyMOL (Version 3.1.1) showed direct contacts with both Cyclin D1 and CDK4 residues at the Site 2 interface, including Arg26/8 and Ala30/12 on the Cyclin D1 α1-helix surface and Phe63/288, His65/290, Phe127/349, Ala130/352, and Asn131/353 on the CDK4 side. In addition to the key residues defined by P2Rank in Table 2, Cyclin D1 Arg29/11 was observed as a recurrent surface-contact residue in two of three replicates. Throughout the manuscript, residues are reported as the canonical UniProt number/MD-composite number; for example, in Phe127/349, 127 is the canonical UniProt number and 349 is the MD-composite number in the combined topology. The complete numbering map is openly available at https://github.com/bkurt00/CDK4/blob/main/Table_S1_residue_mapping.csv (accessed on 19 June 2026).

Among the Cyclin D1 residues listed by P2Rank for Site 2, Lys33/15 was not observed as a recurrent direct contact throughout the Ligand 020 MD trajectory. In contrast, Cyclin D1 Arg29/11, which was not included in the static P2Rank key-residue list, emerged as an additional recurrent contact residue during MD.

### 2.5. MM-GBSA Binding-Free-Energy Profile of Ligand 020

**Figure 4:**
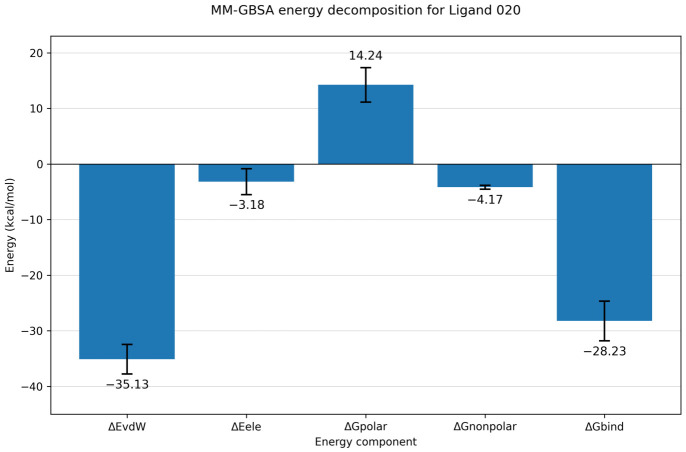
MM-GBSA energy decomposition of Ligand 020 binding at Site 2. Bars show the mean energy components calculated from 102 trajectory frames, with error bars representing standard deviations. The total binding free energy excludes the entropic contribution.

Energy components of the Ligand 020–ternary complex interaction were calculated using the GB^OBC2 model (igb = 5, mbondi2 radii, 0.150 M salt concentration) from the 500 ns trajectory (Table 4 and Figure 4). The total binding free energy, excluding the entropy term (TΔS), was ΔG_bind = −28.23 ± 3.57 kcal/mol (mean ± standard deviation; standard error of the mean = 0.35 kcal/mol).

The van der Waals term (ΔE_vdW = −35.13 kcal/mol) was the largest favorable component of the total binding free energy, whereas the gas-phase electrostatic contribution (ΔE_ele = −3.18 kcal/mol) was comparatively small. The polar-solvation term was unfavorable (ΔG_polar = +14.24 kcal/mol), while the nonpolar-solvation contribution was favorable (ΔG_nonpolar = −4.17 kcal/mol).

### 2.6. Negative-Control Simulations with Clinical CDK4/6 Inhibitors

Palbociclib and ribociclib were forcibly aligned to the Site 2 coordinates and subjected to independent 50 ns simulations under the same OPC/ff19SB/GAFF2/HMR protocol. Both clinical inhibitors lost more than 85% of their initial contacts within the first 3–5 ns of simulation: native contact counts decreased from 828 to 47 for palbociclib (5.7% retention, t = 5 ns) and from 520 to 77 for ribociclib (~14.8% retention, t = 5 ns) (Table 5). After 5 ns, palbociclib maintained a ligand RMSD of approximately 5 Å while skimming over the pocket surface, whereas ribociclib detached around t ~ 15–20 ns and reached a partial-unbinding regime with a ligand RMSD of 7.4 ± 0.7 Å during the final 20 ns.

Under the same protocol, both clinical inhibitors produced more negative ΔG_bind values than Ligand 020 (−28.23 kcal/mol): −33.55 ± 3.61 kcal/mol for palbociclib and −32.76 ± 4.17 kcal/mol for ribociclib. The corresponding van der Waals terms were −47.20 kcal/mol for palbociclib and −43.03 kcal/mol for ribociclib (Table 5).

### 2.7. Reproducibility of Site 2 Engagement Across Independent Replicates

To assess the reproducibility of the Ligand 020 binding mode beyond the single production trajectory, three independent 500 ns replicates (R1–R3) were generated with randomized initial velocities (Section 4.5). Across all three replicates, the ligand never fully dissociated from the Site 2 region: no replicate reproduced the large-scale unbinding (ligand RMSD > 10 Å, solvent escape) observed for the rejected candidates in Table 3. The replicates instead sampled a range of interface-bound substates (Figure S1A). R1, the main-text production trajectory, remained locked in a narrow band (heavy-atom RMSD ≈ 3.5 Å). R2 retained an interface-associated regime through most of the trajectory and showed late-phase excursions toward ≈8 Å without full solvent dissociation. R3 underwent an early metastable repositioning to an ≈5 Å plateau that was then maintained. This behavior is consistent with binding at a shallow, flexible PPI interface rather than a deep occluded cavity, indicating a specific but conformationally plastic Site 2 engagement.

The contact-retention analysis reinforced this interpretation (Figure S1C). Of the 18 interface residues contacted by Ligand 020, 11 recurred as direct contacts in all three replicates. Three residues—Cyclin D1 Arg26/8 and CDK4 Arg123/345 and Phe127/349—were present across every replicate, although their per-residue occupancy varied between replicates. We therefore report these as recurrent interface-contact residues rather than as fixed high-occupancy anchors; the variability in occupancy is itself a signature of the plastic engagement expected at this interface and is consistent with the central premise of this study, namely, that contact-network recurrence is more informative than any single rigid pose or score at PPI surfaces.

Contact analysis of R2 confirmed that the higher RMSD values reflect conformational plasticity within the interface rather than nonspecific surface drifting, as the ligand maintained sustained engagement with the core interaction network (e.g., Arg123/345, Arg26/8, and Phe127/349).

The per-replicate MM-GBSA values were consistent between R1 and R2 (ΔG_bind ≈ −28.2 and −28.8 kcal/mol, respectively), whereas R3 returned a substantially more negative value (≈−64.8 kcal/mol; Figure S1B). This R3 value was not interpreted quantitatively. In R3, the ligand repositioned toward the Cyclin D1 Arg26/8 guanidinium, whose contact occupancy rose to 95% (versus 17% and 36% in R1 and R2). This was accompanied by a selective increase in the gas-phase electrostatic component (ΔE_ele = −29.6 ± 12.9 kcal/mol in R3, versus −3.2 ± 2.3 and −5.0 ± 4.1 kcal/mol in R1 and R2) that was not offset by the polar-solvation term (ΔE_GB = +10.0 kcal/mol), whereas the van der Waals component remained comparable across all replicates (−35.1, −28.8, −40.1 kcal/mol). The R3 over-stabilization was therefore localized entirely to the implicit-solvent electrostatic treatment of a single high-occupancy charged surface contact, consistent with the known tendency of MM-GBSA to over-stabilize salt-bridge-like interactions involving exposed charged residues. In the explicit-solvent trajectory, this residue showed transient Na^+^ coordination, indicating that the ionic screening present in the explicit model is not reproduced by the implicit GB^OBC2 treatment. Taken together, the three replicates support a reproducible conclusion at the level that matters for this target: Ligand 020 maintains persistent engagement with the CDK4/Cyclin D1 Site 2 interface and re-forms a recurring contact network across independent simulations, even though the precise pose is plastic. This is the behavior expected for a genuine but shallow PPI-interface binder and is distinct from the rapid, irreversible unbinding seen for the rejected candidates and for the forced-placement clinical controls (Section 2.2 and Section 2.6). The per-replicate raw data underlying Figure S1 (ligand/backbone/Cα RMSD time series, MM-GBSA decomposition files, and contact-retention tables for R1–R3) are openly available at https://github.com/bkurt00/CDK4/tree/main/replicates (accessed on 19 June 2026).

## 3. Discussion

### 3.1. Screening Efficiency and Dynamic Filtering Rationale

The RapidFunnel-AI/Q-Fold workflow functioned as a practical triage architecture rather than a single-score ranking procedure. The internal Tanimoto cutoff (≥0.30) was applied as an operational threshold to retain a tractable scaffold-hopping candidate set for downstream docking, not as an optimized biological activity threshold. Subsequent ADMET/PAINS/Lipinski filtering, multipocket docking, and MD validation narrowed the search to a single dynamically persistent candidate. This staged narrowing is particularly important for PPI-interface screening because shallow surface pockets can generate many plausible static docking poses that do not survive explicit-solvent dynamics [5].

The early-termination strategy adopted for unstable complexes was not only a computational shortcut but also a decision criterion. Candidates that rapidly drifted from the pocket failed the dynamic barrier required for downstream interpretation, even when short-lived surface contacts or favorable energy terms were present. In this sense, docking was used to generate hypotheses, whereas MD-based pose persistence was used to test whether those hypotheses were physically plausible. Although MM-GBSA calculations were performed for the rejected candidates as well and were openly deposited in the project GitHub repositories (Table 3), they are not discussed in the main text because enthalpy-based scoring is not interpretable for complexes that lose the RMSD < 4 Å structural-stability threshold at an early stage; negative energies in such cases can arise from nonspecific transient surface contacts and, in the absence of structural locking, evaluating the energy component alone increases the risk of false positives.

#### Identity and Known Biological Activities of Ligand_020

**Figure 5:**
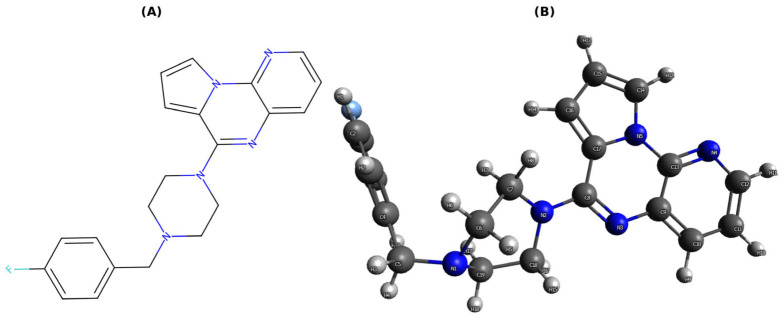
Chemical structure of Ligand_020 (CHEMBL52625; C_21_H_20_FN_5_, MW 361.42). (**A**) 2D chemical structure. (**B**) 3D ball-and-stick representation with atom labels. SMILES: Fc1ccc(CN2CCN(c3nc4cccnc4n4cccc34)CC2)cc1.

Ligand_020 (Figure 5) (ID: CHEMBL52625; C_21_H_20_FN_5_, MW 361.42) is a fluorobenzyl-piperazine derivative bearing a fused pyrrolo-naphthyridine core. The compound’s structural data are publicly deposited in SDF (https://github.com/bkurt00/CDK4/blob/main/ligands/Ligand_020.sdf (accessed on 19 June 2026)) and MOL2 (https://github.com/bkurt00/CDK4/blob/main/ligands/Ligand_020_1.mol2 (accessed on 19 June 2026)) formats, and its SMILES (Fc1ccc(CN2CCN(c3nc4cccnc4n4cccc34)CC2)cc1) is listed as entry #22 in the ADMET-filtered screening library (https://github.com/bkurt00/CDK4/blob/main/cdk4_admet_passed.csv (accessed on 19 June 2026)).

In the literature [12], CHEMBL52625 has been characterized as a serotonin receptor ligand with potent and selective affinity for the 5-HT_3_ receptor at low-nanomolar concentrations, while showing negligible binding across five other serotonin subtypes (5-HT_1a_, 5-HT_1_B, 5-HT_1_D, 5-HT_2a_, 5-HT_2_C) (https://www.ebi.ac.uk/chembl/explore/compound/CHEMBL52625 (accessed on 19 June 2026)). No CDK4-related bioactivity has been previously reported for this compound. In the present study, Ligand_020 was identified through the RapidFunnel-AI pipeline solely based on its predicted structural complementarity to the CDK4/Cyclin D1 PPI interface (Site 2), independent of its known serotonergic profile. The identification of a serotonin-targeted compound as a candidate CDK4/Cyclin D1 modulator is consistent with the principle of polypharmacology, whereby a single molecular scaffold can engage unrelated targets through distinct binding modes.

### 3.2. Structural Stability Versus Static Docking Scores

The rejected ChEMBL candidates illustrate the limitation of relying on static docking-derived poses at PPI interfaces. Ligands 013, 021, and 023 retained protein structural integrity but lost ligand-specific pocket occupation, indicating that receptor RMSD stability alone is insufficient to define a stable complex. For Ligand 021, the close similarity between ligand RMSF (8.08 Å) and ligand RMSD (8.9 Å) is particularly informative: rather than fluctuating around its own equilibrium position, the molecule drifted over the protein surface as an integrated rigid body, reflecting global drift rather than local flexibility.

In contrast, Ligand 020 (Figure 5) reached a persistent RMSD plateau without progressive escape. Although its heavy-atom RMSD (3.56 ± 0.28 Å) was higher than values often expected for deeply buried ATP-pocket inhibitors (typically 1.5–2.5 Å for Site 1), Site 2 is not a deep and closed cavity but a relatively shallow protein–protein interface pocket formed between the CDK4 αE-helix (Arg123–Asn131) and the Cyclin D1 α1-helix (Arg26–Lys33). In this regime, a 3.0–4.0 Å RMSD plateau is characteristic of small-molecule binding in PPI pockets and is consistent with rigid-body-like oscillation of the ligand within the pocket. The homogeneous distribution of ligand-atom RMSF (all 27 heavy atoms within 2.82–3.57 Å) reinforces this interpretation: the ligand oscillated as an integrated unit without fragmentation or conformational collapse, in clear contrast to the heterogeneous RMSF profiles observed in rejected candidates and associated with surface skimming.

### 3.3. Site 2 as a Dynamic PPI-Interface Binding Pocket

The comparison between P2Rank-defined Site 2 residues and the MD contact profile supports the use of Site 2 as a dynamically testable allosteric interface pocket. Ligand 020 contacted residues from both the Cyclin D1 α1-helix surface and the CDK4 αE-helix region, matching the intended PPI-interface targeting strategy. Visual inspection of the final-frame contact network showed that Ligand 020 does not adopt a random placement within Site 2; rather, it establishes a consistent binding mode through the key residue network predicted by P2Rank.

At the same time, the emergence of Cyclin D1 Arg29/11 as a recurrent contact and the absence of persistent Lys33/15 contact show that static pocket prediction and dynamic contact persistence do not need to match residue-by-residue. For predicted PPI pockets, this partial mismatch is expected and reinforces the need for MD validation after pocket prediction. This independent validation point also addresses concerns about possible mismatch between static structure-based pocket predictions and long-timescale MD simulations.

Although crystallographic data confirm that the Site 2 contact residues are located at the CDK4/Cyclin D1 interface [13,14], and previous mutagenesis- and peptide-based studies support the broader functional importance and targetability of this interface [15,16], no published site-directed mutagenesis or biochemical study has individually assessed the contribution of the specific Site 2 residues labeled in Figure 3—Cyclin D1 Arg26/8 and Ala30/12, and CDK4 Arg123/345, Asp126/348, Phe127/349, and Ala130/352—to protein–protein complex formation or stability. In addition, to the best of our knowledge, no experimentally resolved small-molecule or fragment ligand has previously been reported for this specific CDK4 αE-helix/Cyclin D1 α1-helix interface pocket. Therefore, Ligand_020 should be interpreted as a computationally prioritized putative allosteric hit for an underexplored PPI-interface region. Future orthogonal studies, such as alanine-scanning mutagenesis, surface plasmon resonance, isothermal titration calorimetry, or structural characterization, could further support the functional relevance of this pocket and the proposed binding mode of Ligand_020.

### 3.4. Energetic Interpretation and the Role of Negative Controls

The MM-GBSA profile of Ligand 020 was dominated by van der Waals stabilization, with a comparatively small gas-phase electrostatic contribution and an unfavorable polar-solvation penalty balanced by a favorable nonpolar-solvation term. This energetic pattern is consistent with binding at a hydrophobic PPI-interface surface and differs from the typical ATP-pocket recognition mode of clinical CDK4/6 inhibitors, which depends strongly on hinge-region hydrogen bonding and electrostatic complementarity. The polar-solvation penalty (ΔG_polar = +14.24 kcal/mol) reflects the expected electrostatic desolvation cost accompanying transfer of the ligand from bulk solvent into the pocket.

The negative-control simulations further emphasize that energy values alone are not reliable for this system. Palbociclib and ribociclib produced more negative MM-GBSA values than Ligand 020, yet both lost most of their initial Site 2 contacts within 5 ns and failed to preserve their forced binding modes. The clinical inhibitors formed large van der Waals interaction areas (ΔE_vdW: palbociclib −47.20, ribociclib −43.03 vs. Ligand 020 −35.13 kcal/mol) through surface skimming or broad, inconsistent surface contact, yet none of these interactions translated into binding-mode preservation. These controls demonstrate that favorable enthalpy can arise from nonspecific surface contact area during surface skimming or partial unbinding. The decisive criterion in this study is therefore the simultaneous consistency of structural stability, contact retention, and energetic behavior, rather than the most negative docking or MM-GBSA score.

Taken together, the negative-control simulations provide three independent lines of support: (i) The lack of stable Site 2 binding by palbociclib and ribociclib is conceptually consistent with the palbociclib-insensitivity phenotype reported for the p27-CDK4-Cyclin D1 ternary complex in the literature [14], although the present simulations were performed on the p21-CDK4-Cyclin D1 complex, and p21/p27 differences should be interpreted cautiously; this comparison should therefore be viewed as a negative-control result supporting ligand-specific Site 2 binding by Ligand 020, not as direct mechanistic equivalence between p21 and p27 systems. (ii) Stable binding of Ligand 020 under the same setup shows that the distinguishing factor is ligand-specific binding-mode persistence rather than the simulation protocol. (iii) Two independent forced-misalignment controls demonstrate that MM-GBSA energies can be misleading without structural and contact-retention evaluation. The 50 ns duration of these negative controls was sufficient for the intended early-rejection test because both clinical inhibitors lost more than 85% of their initial Site 2 contacts within the first 5 ns, consistent with the early-termination logic applied to unstable ChEMBL candidates.

### 3.5. Limitations and Future Directions

The main limitations of this study remain the use of single-replica MD at the screening stage—although the final candidate, Ligand 020, was additionally validated with three independent 500 ns replicates (Section 2.7)—and the omission of the entropic term from MM-GBSA calculations. Experimental orthogonal validation—such as CDK4/Cyclin D1 interface-binding assays and cellular-proliferation assays—constitutes a natural next step but falls outside the scope of the present computational study. Accordingly, Ligand 020 should be described as a putative allosteric hit rather than a validated inhibitor. Future work should include orthogonal experimental assays for CDK4/Cyclin D1 interface binding, CDK4 activity modulation, and cellular effects. Additional computational validation with ensemble-based entropy estimates, alchemical free-energy calculations, and broader negative-control panels would further test the robustness of the proposed Site 2 binding mode.

## 4. Materials and Methods

### 4.1. ChEMBL-Based Initial Library and AI-Assisted RapidFunnel-AI Prioritization Workflow

The official chemical-representation file of ChEMBL version 33 (chembl_33_chemreps.txt.gz) was downloaded from the EBI FTP server and, after removal of records with empty canonical_smiles fields, the first 50,000 SMILES strings in CHEMBL_ID order were selected as the initial pool. This raw pool served as a target-agnostic input to the Q-Fold/RapidFunnel-AI prioritization engine, which identified structurally relevant candidates by comparing each molecule against three clinically approved CDK4/6 inhibitors (palbociclib, ribociclib, and abemaciclib) via Tanimoto similarity; molecules below the operational threshold (Tanimoto < 0.30) were not considered candidates at any downstream stage. Because CHEMBL_ID assignment is not explicitly based on the CDK4/Cyclin D1 target class, scaffold class, or chemotype, this input set was treated as a chemically heterogeneous, target-agnostic operational subset rather than as a formally randomized sample. Deterministic ID-ordered selection was preferred over random sampling for two practical reasons: (i) it enables exact reproducibility by independent users without requiring random-seed disclosure, and (ii) ChEMBL bulk-download stability was a practical constraint during data acquisition. The subset was therefore not formally randomized but was considered structurally unbiased with respect to the CDK4/Cyclin D1 screening question. Preprocessing steps such as salt stripping, duplicate removal, stereochemical standardization, molecular-weight filtering, or organometallic filtering were deliberately not applied; the aim was to test the capacity of the machine-learning-based prioritization layer to capture discriminative signal in a raw and heterogeneous chemical space. ChEMBL is a large-scale, manually curated drug-discovery database that provides bioactive small molecules with target-, activity-, and literature-based annotations [17].

To accelerate molecular screening in the context of pharmaceutical hit discovery and make the decision process more interpretable, the RapidFunnel-AI/Q-Fold architecture was used. Rather than reducing candidate molecules to a single docking score, this architecture was designed as a multilayer AI-assisted triage system that makes sequential decisions across topological similarity, electronic suitability, drug-likeness, and dynamic binding persistence. In the present workflow, the first reduction step was the internal Q-Fold/RapidFunnel Tanimoto-based topological scan, which reduced 50,000 ChEMBL molecules to 43 candidates. The system represents molecules as Morgan/ECFP-like topological feature vectors that describe local chemical environments rather than rigid 3D conformations. For each candidate, neighborhood relationships expanding from each central atom were calculated with radius = 2, with the aim of recapturing pharmacophoric motifs present in clinical CDK4/6 inhibitors across different scaffolds [18,19]. In this approach, molecular representation was expressed by the following general formulation:

$$\chi_{\mathrm{molecule}\;}=\sum_{i}f\left(\mathrm{center}\;{atom}_{i},{\;\mathrm{radius}}_{n}\right)$$

where Φ(m) denotes the topological feature vector of molecule m, a_i_ denotes atom centers in the molecule, and ECFP(a_i_, r) denotes the fingerprint contribution of the local chemical environment extending from the relevant atom center to radius r. In this study, r = 2 was used, and the resulting topological vectors were combined with basic physicochemical descriptors such as molecular weight, lipophilicity, TPSA, and the number of rotatable bonds [18].

The electronic-property prediction module within RapidFunnel-AI was used as a Q-Fold Thermo-Core-based [20] surrogate model trained on quantum-mechanical reference properties of small organic molecules. The pharmaceutical purpose of this module was not to generate absolute DFT energies for large drug-like molecules, but to enrich candidates relatively within the same representation space toward electronically more suitable and less reactive profiles. The QM9 dataset is a standard reference source containing calculated electronic and thermodynamic properties for small organic molecules composed of CHONF elements [21]. RapidFunnel-AI reformulated this information so that electronic parameters such as HOMO, LUMO, the HOMO–LUMO gap, and isotropic polarizability could be used as a fragment-based enrichment score. Thus, only a smaller and pharmaceutically more plausible candidate pool prioritized by AI was transferred to more computationally expensive physics-based stages such as docking and MD.

**Figure 6:**
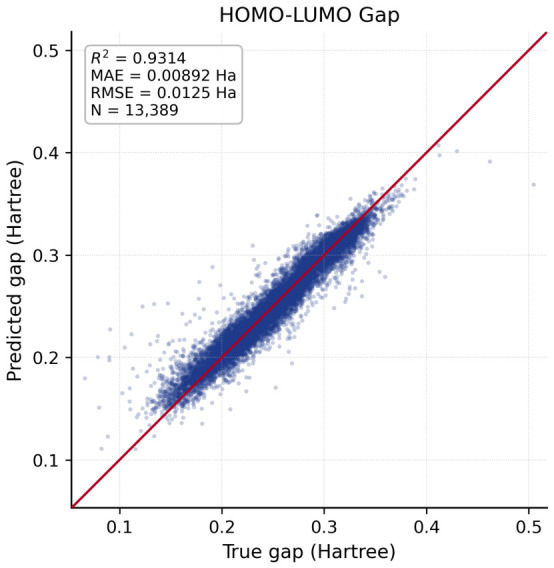
Validation of the HOMO–LUMO gap surrogate model on a random subset of the QM9 dataset (N = 13,389). Predicted values are plotted against DFT-calculated reference values. The model achieves R^2^ = 0.9314, MAE = 0.0089 Ha, and RMSE = 0.0125 Ha, demonstrating sufficient accuracy for electronic enrichment-based candidate prioritization.

**Figure 7:**
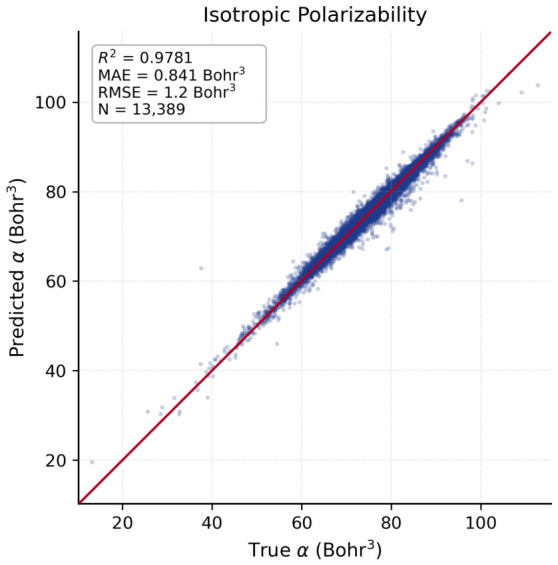
Validation of the isotropic polarizability (α) surrogate model on a random subset of the QM9 dataset (N = 13,389). The model achieves R^2^ = 0.9781, MAE = 0.841 Bohr^3^, and RMSE = 1.2 Bohr^3^, supporting its use as a molecular polarizability-based enrichment criterion in candidate prioritization.

**Figure 8:**
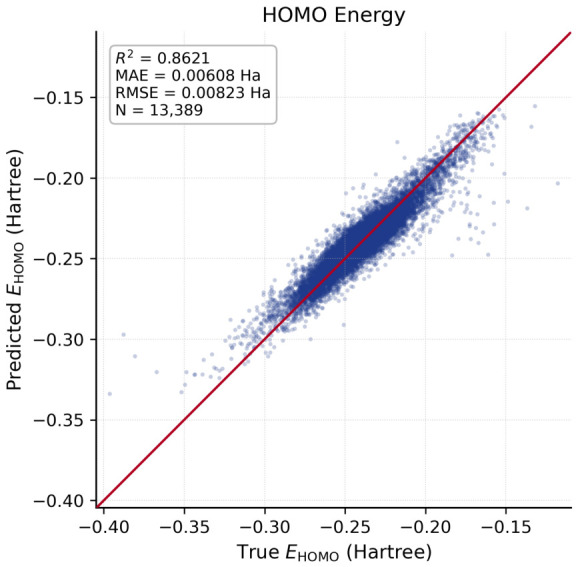
Validation of the HOMO energy surrogate model on a random subset of the QM9 dataset (N = 13,389). The model achieves R^2^ = 0.8621, MAE = 0.00608 Ha, and RMSE = 0.00823 Ha. As a constituent of the HOMO–LUMO gap, HOMO energy predictions serve as an internal consistency check for the electronic enrichment module.

**Figure 9:**
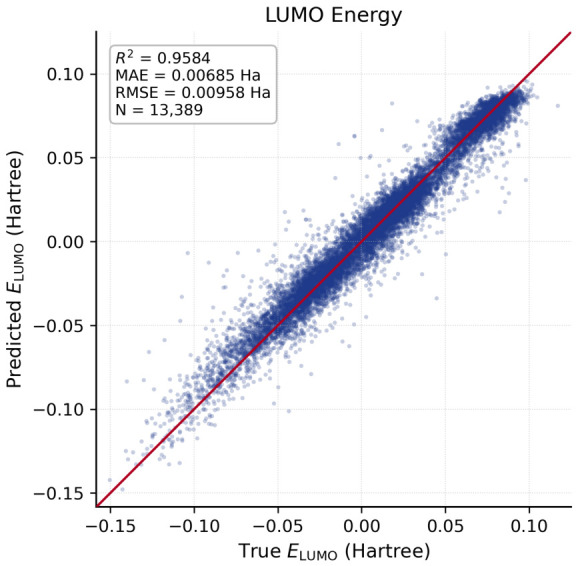
Validation of the LUMO energy surrogate model on a random subset of the QM9 dataset (N = 13,389). The model achieves R^2^ = 0.9584, MAE = 0.00685 Ha, and RMSE = 0.00958 Ha. Together with HOMO energy (Figure 8), LUMO predictions cross-validate the gap-based screening criterion (Figure 6), confirming internal consistency of the electronic surrogate module.

For clarity, the RapidFunnel-AI/Q-Fold prioritization stage should be interpreted as a single internal candidate-prioritization engine rather than as separate post hoc filters. Figure 6, Figure 7, Figure 8 and Figure 9 validate the surrogate models used by this prioritization layer; therefore, they are presented in the methodological description rather than in the downstream Results section. The internal logic of the prioritization stage consisted of the following components: 

(i) Internal Tanimoto/Topological Scan: Candidate molecules were compared against three clinically approved CDK4/6 inhibitors (palbociclib, ribociclib, and abemaciclib) using Morgan/ECFP-type fingerprints and the Tanimoto similarity coefficient. The aim was not to identify generic analogs but to detect scaffold-hopping candidates that contain local chemical motifs associated with clinically effective CDK4/6 inhibitor chemotypes within different scaffold architectures [22]. The Tanimoto threshold (≥0.30) was used as an operational parameter inside the Q-Fold/RapidFunnel prioritization layer to retain a docking-tractable but chemically nontrivial candidate set. It should not be interpreted as a statistically optimized biological activity cutoff.

(ii) Electronic-Property Context within the Same Framework: The Q-Fold Thermo-Core surrogate model described above generates HOMO, LUMO, HOMO–LUMO gap, and isotropic-polarizability predictions for candidate molecules. This module was used to provide relative electronic enrichment and characterization within the same representation space—not to generate absolute quantum-chemical values for large drug-like molecules, and not as a separate 43-to-25 filtering stage.

(iii) Practical Threshold Calibration: The Tanimoto cutoff was selected to balance scaffold-hopping breadth against downstream computational feasibility, yielding 43 candidates from the raw 50,000-molecule ChEMBL subset. The subsequent reduction from 43 to 25 candidates was performed by ADMET/PAINS/Lipinski filtering, as summarized in Table 1. The per-ligand chemical basis of each similarity assignment, including shared motifs and interpretation strength, is available at https://github.com/bkurt00/CDK4/blob/main/QFold_RapidFunnel_Chemical_Interpretability_Tables.docx (accessed on 19 June 2026).

Candidates passing the Q-Fold/RapidFunnel prioritization stage were then subjected to an ADMET-oriented filter. Using RDKit, candidates were filtered according to the following criteria: at most one Lipinski violation, TPSA ≤ 140 Å^2^, number of rotatable bonds ≤ 10, and zero PAINS alerts [18,19,20,21,23,24]. Lipinski’s rules assess oral drug-likeness in terms of molecular weight, lipophilicity, and numbers of hydrogen-bond donors and acceptors [24], whereas the QED score summarizes drug-likeness through a multiparameter desirability function [25]. PAINS alerts were evaluated to exclude pan-assay interference structures that may generate false positives in screening studies [23]. The 25 candidates passing ADMET/PAINS/Lipinski filtering were ranked by QED score and transferred to the structure-based evaluation phase, namely, docking.

The final pool of candidate ligands passing the ADMET filter, the RDKit-based script that converts SMILES inputs into 3D SDF format, and the 3D coordinate files of the ligands were shared openly in the project repository. The final candidate pool is available in the cdk4_admet_passed.csv file at https://github.com/bkurt00/CDK4/blob/main/cdk4_admet_passed.csv (accessed on 19 June 2026), and the SMILES-to-3D conversion workflow is provided in the convert_smiles2sdf.py script at https://github.com/bkurt00/CDK4/blob/main/convert_smiles2sdf.py (accessed on 19 June 2026). This script uses the RDKit ETKDGv3 algorithm for 3D conformer generation and the MMFF94 force field for geometry optimization.

Ligand numbering (Ligand_000–Ligand_024) corresponds to the row order in the post-ADMET CSV file. All ligand files used in the study and converted into 3D format are provided collectively in the https://github.com/bkurt00/CDK4/tree/main/ligands (accessed on 19 June 2026) directory. Thus, the ligand identities reported in the docking, MD, and contact-analysis stages can be directly matched to the source SMILES inputs.

#### 4.1.1. Independent Representativeness Control of the Selected Subset

To address the concern that CHEMBL_ID-ordered selection may introduce uncontrolled chemotype bias, an internal consistency analysis was performed. The 50,000-molecule subset was split into two non-overlapping halves by ID order (entries 1–25,000 and 25,001–50,000), and six drug-relevant molecular descriptors were computed for each molecule using RDKit: molecular weight, Wildman–Crippen LogP, number of hydrogen-bond acceptors and donors, topological polar surface area, and heavy-atom count. The descriptor distributions of the two halves were compared using two-sample Kolmogorov–Smirnov tests (https://github.com/bkurt00/CDK4/blob/main/chembl_representativeness/chembl_50k_representativeness.png (accessed on 19 June 2026)). All pairwise KS statistics remained below 0.06 (range: 0.014–0.059), indicating negligible practical divergence despite formal statistical significance at large sample sizes (N = 25,000 per group). The two halves produced nearly superimposable distribution profiles across all six descriptors, confirming that CHEMBL_ID ordering does not impose systematic clustering in molecular-property space. Scaffold diversity was assessed through Murcko generic decomposition: 12,423 unique scaffolds were identified among 49,998 parseable molecules (diversity ratio = 0.249), demonstrating broad structural heterogeneity within the operational subset. Of the 49,998 molecules, 33,598 (67.2%) satisfied all four Lipinski criteria, consistent with the expected profile of an unfiltered ChEMBL extract that includes peptides, natural products, and other non-oral-drug-like chemotypes. These results support the use of the ID-ordered subset as a chemically heterogeneous, target-agnostic input pool for the downstream RapidFunnel-AI/Q-Fold prioritization pipeline. The analysis script, raw SMILES input, descriptor statistics, and the distribution figure are available at https://github.com/bkurt00/CDK4/tree/main/chembl_representativeness (accessed on 19 June 2026).

#### 4.1.2. Retrospective Clinical Phase Analysis of the Filtered Candidates

To evaluate the stringency of the applied ADMET/PAINS filters, and to ensure that approved drugs or clinically annotated candidates were not inadvertently discarded, a retrospective cross-reference was conducted using the ChEMBL API based on the max_phase annotation. The 18 candidates eliminated during the ADMET stage and the final 25 retained ligands were analyzed. The eliminated compounds all returned max_phase = 0, indicating that none were approved drugs or clinical candidates. Similarly, the final 25 retained ligands also consisted entirely of max_phase 0 compounds. These results confirm that, in this specific screening campaign, the heuristic filtering step did not inadvertently remove any approved drugs or annotated clinical candidates. The analysis script and corresponding data subsets are openly available in the project repository under the admet_phase_control directory: https://github.com/bkurt00/CDK4/tree/main/admet_phase_control (accessed on 19 June 2026).

### 4.2. Method Reproducibility and Data Availability

To increase the transparency and pharmaceutical reusability of the AI-prioritization component of the pipeline, the Q-Fold module of the RapidFunnel-AI architecture has been made available for academic use as a web server at https://qfold.biochemoinfo.com (accessed on 19 June 2026). This server was designed not only for sharing results but also for re-querying the decision process: users can upload SMILES inputs and reproduce topological matching, electronic-property prediction, and prioritization scores within the same decision logic. Thus, this study moves beyond a one-off closed in silico screening and provides a reusable AI-assisted pharmaceutical-discovery platform for rapid triage of new candidates against PPI-focused targets.

### 4.3. Binding-Site Prediction, Ligand Preparation, and Molecular Docking

The PDB ID: 6P8H crystal structure was used as the structural template for the CDK4/Cyclin D1 system because of its high sequence homology [26]. Candidate molecules passing the RapidFunnel-AI and ADMET-like filtering stages were subjected to structure-based molecular-docking evaluation on the CDK4/Cyclin D1 complex. The purpose of the docking stage was not to focus exclusively on the canonical ATP-binding pocket but also to evaluate ligandable regions on the surface of the CDK4/Cyclin D1 complex, particularly those near the protein–protein interface. Therefore, target regions were not restricted to a single catalytic pocket; putative pockets with high ligand-binding potential were identified using PrankWeb/P2Rank [27,28].

Docking studies used the same (6P8H) crystal structure. Four putative binding regions identified by PrankWeb/P2Rank analysis were defined as docking-grid centers. The coordinates and biological relevance of these regions are presented in Table 2. For reproducibility, the PrankWeb data used in this study are available as a GitHub repository at https://github.com/bkurt00/CDK4/tree/main/prankweb-6P8H (accessed on 19 June 2026).

The structural identities of the four pockets identified by P2Rank were evaluated according to the positions of their residues within the CDK kinase architecture (Table 2). Pocket 1 was defined as the canonical ATP-binding pocket because it contains the β3-strand catalytic lysine (Lys35), the hinge region (Phe90-Glu91-His92), and the DFG motif (Asp155-Phe156); this pocket corresponds to the target pocket of clinically approved inhibitors such as palbociclib, ribociclib, and abemaciclib. Pocket 2 is a mixed pocket containing residues from both CDK4 (chain B) and Cyclin D1 (chain A), is located at the CDK4/Cyclin D1 protein–protein interface, and constitutes the primary pocket of interest for the allosteric-targeting strategy of this study. Pocket 3 is located away from the ATP pocket in a secondary interface region. Pocket 4 is a marginal surface depression with a low confidence score (probability = 0.005) and was included in the pipeline for comparison.

The multipocket approach was used to evaluate the binding potential of candidate ligands not only to the ATP-binding pocket but also to the CDK4/Cyclin D1 interface and nearby alternative surface pockets. Site 2 was not manually imposed as the final binding site of Ligand 020 after docking. Instead, it was one of four ligandable pockets independently predicted by PrankWeb/P2Rank; it was biologically prioritized because of its location at the CDK4/Cyclin D1 PPI interface, whereas Ligand 020 emerged as the only candidate that maintained a persistent binding mode there after docking and MD validation.

During ligand preparation, the protonation, tautomeric, stereoisomeric, and ring-conformation states of candidate molecules passing the ADMET filter were evaluated under conditions close to physiological pH; Gypsum-DL was used to generate these variants [29]. Ligands were prepared at pH 7.4, and a limited number of possible variants were generated for each compound. Ligand structures generated from SMILES inputs were subjected to hydrogen addition, 3D conformer generation, and geometry optimization with RDKit; the ETKDGv3 algorithm was used for 3D conformer generation; and the MMFF94 force field was used for geometry relaxation when applicable. The ligands were then converted into PDBQT format (example code summarizing the ligand-preparation procedure is here: https://github.com/bkurt00/CDK4/blob/main/prepare_ligand_example.py (accessed on 19 June 2026); SDF and MOL2 formats of all ligands used in the manuscript are in the following repository folder: https://github.com/bkurt00/CDK4/tree/main/ligands (accessed on 19 June 2026)).

During receptor preparation, water molecules were removed from the crystal structure, and atom-type conversions required for docking calculations were performed. Depending on the docking scenario, existing heteroatom/ligand records were either retained or removed. In receptor PDBQT conversion, atom types were assigned in an AutoDock/Vina-compatible format, and simple structural-control steps were applied to reduce conversion errors that could compromise structural integrity.

### 4.4. Hydrated and Dry Docking Procedures

Docking was performed using two independent and parallel protocols. The 25 candidate ligands passing the ADMET filter were simultaneously submitted to both a Vina-GPU-based [30,31] dry docking workflow and an AutoDock-GPU-based [32,33] hydrated docking workflow. The purpose of this approach was to provide independent evaluation by two different scoring functions (Vina and AutoDock4) and two different levels of solvent representation (without explicit solvent versus hydration-mediated poses); the protocols functioned as cross-validation workflows rather than as prescreens for one another.

In Vina-GPU calculations, search depth = 8, num_modes = 9, and exhaustiveness = 32 were used. Solvent molecules were not represented explicitly, and scores were interpreted within the Vina scoring function [30,31]. In AutoDock-GPU calculations, the hydration option was used during ligand preparation; binding scores were calculated from AutoDock4-based grid/energy maps with a grid spacing of 0.375 Å, 50 independent docking runs, and 10,000,000 energy evaluations.

In both workflows, the four pocket centers defined by PrankWeb/P2Rank (see Section 4.3) were used separately as docking-grid centers. To avoid repeatedly counting similar poses, a duplication check based on the distance between pose centers (3.0 Å threshold) was applied; poses near grid boundaries were re-evaluated, when necessary, using an expanded grid with a maximum size of 45.0 Å.

Dry and hydrated docking results were not interpreted as outputs on the same scoring scale. Because these approaches use different scoring functions, different grid/energy terms, and different levels of solvent representation, docking scores were used not as absolute binding free energies but for relative ranking and pose selection within each protocol. Final evaluation of candidates was based not only on score magnitude but also on whether the binding pose remained within the target pocket, its proximity to the CDK4/Cyclin D1 interface, its non-ATP-pocket positioning, and its structural stability in subsequent molecular-dynamics simulations. The most stable poses obtained from both workflows were transferred to the next stage: molecular-dynamics simulation.

### 4.5. Molecular-Dynamics Simulations and Trajectory Analysis

The best ligand poses obtained from docking outputs were cleaned from PDB/PDBQT formats, AutoDock/Vina-specific records were removed, and ligand structures were converted into MOL2 format suitable for MD parameterization. Docking-water records present in hydrated AutoDock-GPU outputs were not transferred directly into the MD systems, so as to avoid compatibility problems during tleap topology generation. Instead, all complexes were subsequently re-solvated using the same explicit-solvent protocol. Net ligand charges were evaluated separately for each ligand according to the protonation states expected under physiological pH conditions [34,35]. The Amber ff19SB force field was used for the protein, GAFF2 for the ligands, and the OPC water model for water [35,36,37]. The use of the OPC water model in this study was based on the recommendation in the Amber24 Manual [34], which emphasizes that although TIP3P is computationally efficient, OPC and OPC3 provide improved electrostatic accuracy. Complexes were solvated with tleap in an OPC explicit-water box with a 10 Å buffer, and systems were neutralized with Na^+^/Cl^−^ ions. After ligand processing with antechamber/GAFF2, missing parameters were generated with parmchk2, and solvation and ion neutralization were carried out in tleap using ff19SB, GAFF2, and OPCBOX; topology and coordinate files were then saved.

After Amber topology files were generated, hydrogen-mass repartitioning was applied, and HMR-compatible topologies were prepared using the hmassrepartition approach in ParmEd [38], enabling the use of a 4 fs time step. Production simulations were run with the same OpenMM (Version 7.0) protocol [39], and trajectory files were saved in NetCDF format. For the final candidate (Ligand 020), three independent 500 ns replicates were generated by reinitializing atomic velocities from the Maxwell–Boltzmann distribution at 300 K using different random seeds, while keeping the minimization, heating, and NPT-equilibration protocol identical. The trajectory reported in the main text (Replicate 1) corresponds to the production run; Replicates 2 and 3 were used to assess the reproducibility of Site 2 engagement (Section 2.7; Figure S1).

Trajectory frames were written every 0.2 ns for most systems and every 0.5 ns for the long 500 ns simulation; energy records were written every 10 ps, and checkpoint files every 0.1 ns, with only the last five checkpoints retained. Systems were built with PME electrostatics, a 10 Å non-bonded cutoff, an 8 Å switching distance, HBonds constraints, and rigid-water settings. Simulations used the LangevinMiddle integrator; temperature was set to 300 K, the friction coefficient to 1 ps^−1^, and pressure to 1 bar. Pressure was controlled with a Monte Carlo barostat.

Before production simulation, each system underwent energy minimization, heating, and equilibration. First, a 5000-step energy minimization was performed, after which the system was heated from 1 K to 300 K over 1 ns using a 10-stage protocol. This was followed by a 1 ns NPT equilibration stage. The production script defined minimization, heating, NPT equilibration, NetCDF trajectory writing, and checkpoint-validation steps.

Trajectory analyses were performed using cpptraj, MDTraj, MDAnalysis [40,41,42], and an AmberTools-based analysis workflow. Before analysis, trajectory files were concatenated, recentered according to the protein center of mass, and imaged to keep the ligand near the protein, thereby reducing periodic-boundary-condition artifacts. Protein Cα RMSD, backbone RMSD, ligand heavy-atom RMSD, ligand RMSF, and Cα RMSF were calculated. For ligand RMSD calculations, structures were first aligned to protein Cα atoms, and then no-fit RMSD values were calculated for ligand heavy atoms. The analysis script explicitly defines calculations for protein Cα RMSD, backbone RMSD, ligand RMSD, and RMSF. Native contacts were defined as ligand heavy atom–protein heavy atom pairs within 4.0 Å in the initial post-equilibration frame; contact retention was computed as the fraction of these initial contact pairs present in each subsequent frame and is reported in Table 5 for the negative-control simulations.

MM-GBSA analysis was performed for candidates that reached this stage to evaluate binding-free-energy trends. Before MM-GBSA calculations, solvent and ions were stripped from the trajectory, and complex, receptor, and ligand topologies were prepared using ante-MMPBSA.py. Calculations were performed with MMPBSA.py [43]. The mbondi2 radii, the igb = 5 GB model, and a salt concentration of 0.150 M were used. Approximately 100 frames were targeted from the trajectory, and the first 10% was excluded to focus on post-equilibration stability. MM-GBSA results were interpreted not as absolute experimental binding free energies but as relative energetic trends among candidates obtained under the same protocol [44]. The entropy term (TΔS) was not included. Because entropy estimation (quasi-harmonic or normal-mode analysis) is computationally demanding and sensitive to structural-quality conditions (rigid snapshots versus ensemble averaging), this study used MM-GBSA for enthalpy-based relative ranking. The effects of entropy contributions are discussed in the Discussion section.

As negative-control simulations within the same protocol, two clinically approved CDK4/6 inhibitors, palbociclib and ribociclib, were manually aligned into the Site 2 pocket, where Ligand 020 had emerged as the only dynamically persistent candidate after the multipocket docking and MD-validation workflow, and subjected to independent 50 ns MD simulations. Abemaciclib was also evaluated as a clinical reference compound during the multipocket docking-control stage; however, it did not produce a retained and physically meaningful docking pose in any of the four PrankWeb/P2Rank-defined pockets under the same dry and hydrated docking criteria. This forced-placement setup was therefore used to test whether the Site 2 binding regime was specific to Ligand 020 or could also be maintained by ATP-pocket clinical inhibitors. Ligand preparation (Gypsum-DL, ETKDGv3, MMFF94), parameterization (GAFF2 + parmchk2), system setup (ff19SB + OPC explicit-water box, 10 Å buffer, Na^+^/Cl^−^ neutralization), HMR application (4 fs time step), minimization, heating, NPT equilibration, production settings (PME, LangevinMiddle integrator, Monte Carlo barostat), and the analysis pipeline (RMSD, RMSF, native-contact profile, MM-GBSA) were identical to those used for the Ligand 020 system. This design ensures that any observed difference arises from ligand properties rather than from the simulation setup.

### 4.6. Methodological Limitations

This study has two main limitations: First, the entropy term (TΔS) was omitted from the MM-GBSA binding-free-energy calculations. Entropy calculation (quasi-harmonic fluctuation analysis or normal-mode analysis) requires careful interpretation, particularly in small ligand–protein systems, because it depends on structural quality, ensemble representativeness, solvation-shell selection, and high computational cost. Therefore, the MM-GBSA results were evaluated not as absolute experimental binding free energies but as relative enthalpic trends within the same protocol. Second, this study does not include experimental binding, enzyme inhibition, or cellular validation. Ligand 020 should therefore be presented at this stage not as a validated inhibitor but as a putative allosteric hit candidate prioritized by AI-assisted triage, multipocket docking, 500 ns MD stability across three independent replicates, contact retention, and MM-GBSA consistency. Future work should validate its pharmaceutical potential using CDK4/Cyclin D1 interface-binding assays, CDK4 activity measurements, cellular-proliferation assays, and, if possible, ensemble-based entropy or alchemical free-energy calculations.

Surrogate Model Applicability Domain and Intentional Use as a Coarse Pre-Filter: The Q-Fold Thermo-Core electronic-property surrogate was trained on the QM9 dataset, which is composed of small CHONF molecules with up to nine heavy atoms, and was then applied to candidate ligands that exceed this size range. We acknowledge this domain mismatch but do not interpret it as a methodological flaw of the present pipeline, because the surrogate was deliberately deployed as a coarse upstream sieve rather than as a quantitative predictor of drug-like electronic properties. Within RapidFunnel-AI, the surrogate effectively performs a fragment-based screen: it captures the local electronic tendencies of substructural motifs shared with the QM9 chemical universe and ranks candidates relatively within the same representation space, without claiming DFT-level accuracy for the full drug-like molecule. This functional role is analogous to keyword-based pre-filtering in large-scale web search, where a fast and approximate filter narrows billions of records down to a manageable shortlist that is then subjected to more rigorous downstream evaluation. In our pipeline, the downstream evaluation consists of orthogonal physics-based stages—multipocket docking with two independent scoring functions, 500 ns explicit-solvent MD simulations, native-contact persistence analysis, and MM-GBSA energy decomposition—which carry the decisive weight in candidate selection. The decision to begin from 50,000 raw ChEMBL molecules without prior cleaning was motivated precisely by this design philosophy: a fast, deliberately coarse AI-assisted sieve at the entry point of the funnel is intended to operate on heterogeneous chemical space and is followed by progressively stricter physics-based filters. We therefore view the QM9-to-drug-like extrapolation not as a hidden weakness of the surrogate but as a controlled trade-off consistent with the sieve-like function that the surrogate is assigned in the architecture. Independent validation of the surrogate on drug-like molecules through dedicated DFT benchmarking remains a worthwhile direction for future work.

To prevent out-of-domain application, the Q-Fold/RapidFunnel web server (https://qfold.biochemoinfo.com (accessed on 19 June 2026)) enforces a built-in applicability domain threshold: for molecules exceeding 9 heavy atoms, electronic-property predictions (HOMO, LUMO, HOMO–LUMO gap, isotropic polarizability) are automatically disabled, and the system returns only topological similarity rankings based on Morgan/ECFP-type fingerprints. Because all 25 ADMET-filtered candidates in the present study exceed this threshold, the electronic surrogate module did not contribute to their ranking or selection; the downstream candidate evaluation relied entirely on fingerprint-based similarity, multipocket docking, explicit-solvent MD, and MM-GBSA analysis. The Q-Fold/RapidFunnel web server was designed from the outset with this hard-coded applicability domain check, ensuring that electronic-property predictions are never returned for molecules outside the QM9 chemical space on which the surrogate models were trained.

## 5. Conclusions

This study presents a multistage pharmaceutical-discovery pipeline for identifying putative allosteric small-molecule candidates targeting the CDK4/Cyclin D1 protein–protein interface, supported by AI-assisted prioritization and validated by physics-based simulations. RapidFunnel-AI/Q-Fold systematically narrowed 50,000 ChEMBL molecules to 43 candidates through an internal Tanimoto-based topological scan derived from clinical CDK4/6 inhibitor motifs. ADMET/PAINS/Lipinski filtering then yielded 25 ligands for multipocket docking, nine complexes were transferred to MD simulation, and the final decision was based on the combined evaluation of explicit-solvent MD, contact retention, and MM-GBSA energy components.

The most important contribution of this approach is showing that docking or MM-GBSA scores alone are insufficient for shallow and flexible targets such as PPI interfaces. Ligand 020 remained stable in the Site 2 pocket for 500 ns; this interface engagement was reproduced across three independent replicates with no full dissociation (Section 2.7). The decisive distinction was therefore not RMSD alone but the joint persistence of the binding mode, native-contact network, and energetically consistent interaction profile. This divergence demonstrates that pharmaceutical hit selection should be based on the joint consistency of decision metrics rather than on score magnitude alone.

Ligand 020, the output of this study, should be regarded not as a validated clinical inhibitor but as an experimentally testable putative allosteric hit candidate targeting the CDK4/Cyclin D1 interface. Moreover, the decision-interpretable and web-server-queryable structure of RapidFunnel-AI/Q-Fold, accessible at https://qfold.biochemoinfo.com, transforms this study from a single-ligand report into a reusable AI–simulation platform for PPI-targeted oncological drug discovery. In this respect, the present study directly contributes to the pharmaceutical drug-discovery literature at the intersection of computational drug discovery, medicinal chemistry, and AI-assisted pharmaceutical hit prioritization.

## Figures and Tables

**Table 1:** Sequential RapidFunnel-AI screening protocol and the number of surviving candidate molecules at each stage.

Stage	Input	Criterion/Decision Logic	Output
Raw ChEMBL library	-	ChEMBL 33 canonical SMILES subset.	50,000
Q-Fold/RapidFunnel-AI prioritization	50,000	Internal Tanimoto/topological scan against palbociclib, ribociclib, and abemaciclib reference motifs; operational cutoff ≥ 0.30 selected to retain a docking-tractable scaffold-hopping candidate set.	43
ADMET/PAINS/Lipinski	43	Max. 1 Lipinski violation; TPSA ≤ 140 Å^2^; rotatable bonds ≤ 10; PAINS = 0.	25
Multipocket docking (dry Vina-GPU + hydrated AutoDock-GPU; 4 pockets)	25	Pose quality; pocket retention; interface proximity; docking-water consistency where applicable; Gypsum-DL protomer/tautomer variants included in the total job count.	9 complexes
MD simulation (≤500 ns; single-replica screening)	9 complexes	RMSD/RMSF stability; ligand drift; contact retention; complex integrity. Final candidate additionally validated by three independent 500 ns replicates (Section 2.7).	1
Final candidate	1	Persistent Site 2 binding mode after docking/MD validation.	Ligand 020

**Table 2:** Ligandable pockets identified by PrankWeb/P2Rank in the 6P8H ternary complex (CDK4/Cyclin D1/p21^Cip1). Residue numbers are reported as UniProt canonical/MD-composite. Chain labels: A = Cyclin D1 (UniProt P24385, residues 19-262), B = CDK4 (UniProt P11802, residues 18-295), C = p21^Cip1 (UniProt P38936, residues 14-67).

Pocket and P2Rank Metrics	Pocket Annotation
Site 1 Coordinates: 19.36, −12.74, −19.02 P2Rank score: 6.65 Probability: 0.368	Key Residues: B (CDK4): Ala33/258, Lys35/260, Leu57/282, Val69/294, Leu71/296, Phe90/315, Glu91/316, His92/317, Glu141/360, Leu144/363, Ala154/373, Asp155/374, Phe156/375, Leu158/377. Motif/Structural Position: Lys35 = beta3 catalytic lysine; Phe90-Glu91-His92 = hinge; Asp155-Phe156 = DFG motif; Leu57-Val69-Leu71 = hydrophobic floor of the ATP pocket. Structural Identity: Canonical ATP-binding pocket of CDK4. Role in This Study: Clinical reference pocket corresponding to the target region of palbociclib, ribociclib, and abemaciclib.
Site 2 Coordinates: 13.26, −23.66, −32.87 P2Rank score: 4.31 Probability: 0.191	Key Residues: A (Cyclin D1): Arg26/8, Ala30/12, Lys33/15. B (CDK4): Phe63/288, His65/290, Arg123/345, Asp126/348, Phe127/349, Ala130/352, Asn131/353. Motif/Structural Position: A: Arg26-Lys33 = Cyclin D1 α1 helix (N-CBF, CDK-binding surface); B: Phe63-His65 = CDK4 beta3-alphaC loop; B: Arg123-Asn131 = C-terminal end of the CDK4 αE helix. Structural Identity: Protein–protein interface pocket formed between the CDK4 αE helix and the Cyclin D1 α1 helix in the ternary complex. Role in This Study: Primary allosteric target pocket at the interface of the palbociclib-refractory ternary complex.
Site 3 Coordinates: 36.33, −0.26, −43.41 P2Rank score: 2.68 Probability: 0.081	Key Residues: A (Cyclin D1): Phe88/70, Pro93/75, Val94/76, Lys95/77, Arg98/80, Leu101/83, Met140/122, Leu143/125. C (p21): Leu30/519, Asp33/522, Cys34/523, Leu37/526. Motif/Structural Position: A: Phe88-Leu101 = periphery of the Cyclin D1 cyclin-box fold, near the RXL groove; A: Met140-Leu143 = cyclin-box C-domain; C: Leu30-Leu37 = p21 KID 3_10-helix region. Structural Identity: Secondary Cyclin D1/p21 interface pocket. Role in This Study: Comparison pocket representing a CDK4-independent protein–protein interaction region.
Site 4 Coordinates: 8.01, −23.18, −14.77 P2Rank score: 0.94 Probability: 0.005	Key Residues: B (CDK4): Leu97/322, Leu117/335, Phe121/340, Ile143/362, Val151/370, Met204/411. Motif/Structural Position: Surface depression near the beta5 strand and αE-loop region; no clear matched functional motif. Structural Identity: Marginal CDK4 surface depression. Role in This Study: Negative-control pocket with very low predicted probability (0.005).

**Table 3:** Dynamic profiles of ChEMBL candidate ligands rejected during the MD-based filtering stage. All simulations were performed with the same protocol used for Ligand 020 (ff19SB/GAFF2/OPC/HMR, 4 fs time step). Protein backbone RMSD values remained within the 2.1–3.5 Å range in all systems, preserving the structural integrity of the active pocket; the reported instabilities are ligand-specific.

Ligand	Pocket/Docking and Duration	Dynamic RMSD/RMSF Profile	Dominant Contacts and Behavior	Rejection Rationale and Data Access
Ligand 013	Site 2/dry 200 ns (1000 frames)	Final-phase ligand RMSD: 6–9 Å in the middle phase; 13.7 Å in the final 250 frames Maximum ligand RMSD: 13.7 Å Maximum ligand RMSF: 6.7 Å	Cyclin D1 Arg26/8: 46% of frames (transient) Structural behavior: progressive unbinding	Complete departure from the active pocket; critical contacts remained transient. Data access: https://github.com/bkurt00/CDK4/tree/main/dry_site2_ligand13 (accessed on 19 June 2026)
Ligand 021	Site 3/dry 200 ns (1000 frames)	Final-phase ligand RMSD: 8.9 Å Maximum ligand RMSD: 8.9 Å (ligand); 7.6 Å (core) Maximum ligand RMSF: 8.08 Å	Dominant contact: surface contacts after drift Structural behavior: surface skimming	Negative energy at RMSD > 8 Å represents an unstable docking-derived pose profile; no structural locking was observed. Data access: https://github.com/bkurt00/CDK4/tree/main/dry_site3_ligand21 (accessed on 19 June 2026)
Ligand 023	Site 2/dry 200 ns (1000 frames)	Final-phase ligand RMSD: 7.5–8.5 Å plateau Maximum ligand RMSD: ~8.5 Å Maximum ligand RMSF: not determined	Cyclin D1 Arg26/8: 47%; CDK4 Ala130/352: 22%; Cyclin D1 Val27/9: 11% Structural behavior: surface sliding	Loss of deep-pocket occupation; only shallow hydrophobic surface contacts were retained. Data access: https://github.com/bkurt00/CDK4/tree/main/dry_site2_ligand23 (accessed on 19 June 2026)
Other candidates (LigandX, LigandY, …)	Site 2/dry and hydrated 20–50 ns (early rejection)	Final-phase ligand RMSD: 5.0–10.0 Å within the first 20–50 ns Maximum ligand RMSD: >5 Å (rapid unbinding threshold) Maximum ligand RMSF: not determined	Cyclin D1 Arg26/8 and CDK4 Phe127/349 contacts were not retained Structural behavior: rapid unbinding and solvent exposure	Early ligand drift failed the RMSD < 4 Å stability criterion; therefore, favorable energies, when present, were not interpreted as evidence of stable binding. Data Access *

* Analysis datasets for eliminated hydrated candidates are available in the project repository under folders prefixed with hydrated_: https://github.com/bkurt00/CDK4 (accessed on 19 June 2026).

**Table 4:** Ligand 020—MM-GBSA binding-free-energy decomposition at Site 2 (CDK4/Cyclin D1/p21 ternary complex). All values are in kcal/mol; standard deviations were calculated over 102 frames. The entropy contribution (TΔS) was not calculated; data are reported for enthalpy-based relative ranking. The mmgbsa.dat file can be found here: https://github.com/bkurt00/CDK4/tree/main/dry_site2_ligand20 (accessed on 19 June 2026).

Component	Mean	Std. Deviation	Std. Error
ΔE_vdW (van der Waals)	−35.13	2.66	0.26
ΔE_ele (gas-phase electrostatic)	−3.18	2.31	0.23
ΔG_polar (EGB, polar solvation)	+14.24	3.09	0.31
ΔG_nonpolar (ESURF, nonpolar solvation)	−4.17	0.35	0.03
ΔG_bind (total, excluding TΔS)	−28.23	3.57	0.35

**Table 5:** Comparison of 50 ns negative-control simulations in the Site 2 pocket (palbociclib, ribociclib) with the 500 ns Ligand 020 simulation. All systems were run with the same OPC/ff19SB/GAFF2/HMR protocol.

Metric	**Ligand 020**	**Palbociclib**	**Ribociclib**
Simulation time	500 ns	50 ns	50 ns
Protein Cα RMSD (mean)	2.88 ± 0.32 Å	3.04 ± 0.59 Å	2.44 ± 0.42 Å
Ligand RMSD (last 10 ns)	3.56 ± 0.28 Å	4.70 ± 0.54 Å	7.42 ± 0.65 Å
Ligand RMSD (max)	4.20 Å	5.77 Å	9.27 Å
Native contact retention (t = 5 ns)	stable plateau	5.7%	14.8%
Structural behavior	continuous plateau	surface drifting	partial unbinding
ΔG_bind (MM-GBSA, kcal/mol)	−28.23 ± 3.57	−33.55 ± 3.61	−32.76 ± 4.17
ΔE_vdW (kcal/mol)	−35.13	−47.20	−43.03
ΔE_ele (kcal/mol)	−3.18	−11.24	−8.47
ΔG_polar (kcal/mol)	+14.24	+30.09	+23.76
Interpretation	stable binding-mode persistence	forced Site 2 misalignment; unstable binding	forced Site 2 misalignment; unstable binding

## Data Availability

The original data presented in the study are openly available in GitHub at https://github.com/bkurt00/CDK4 (accessed on 19 June 2026), including ligand files, ligand-preparation scripts, molecular-dynamics analysis files, RMSD/RMSF time-series data, contact-analysis outputs, MM-GBSA summaries, and residue-numbering tables. The web server used for AI-assisted molecular prioritization, RapidFunnel-AI/Q-Fold, is available at https://qfold.biochemoinfo.com (accessed on 19 June 2026).

## Supplementary Materials

The following supporting information can be downloaded at https://www.mdpi.com/article/10.3390/ph19060970/s1: Figure S1. Replicate trajectory and structural validation dataRMSD/RMSF time series, contact-analysis outputs, MM-GBSA calculation summaries, residue-numbering tables, and analysis scripts are available in the project GitHub repository: https://github.com/bkurt00/CDK4 (accessed on 19 June 2026).
