# Supplementary figures and images for: AI-Assisted Identification of a Putative Allosteric Ligand Targeting the CDK4/Cyclin D1 Protein–Protein Interface
Source: Pharmaceuticals (Basel). 2026 Jun 22;19(6):970. doi: 10.3390/ph19060970 (PMC13305350; doi:10.3390/ph19060970)

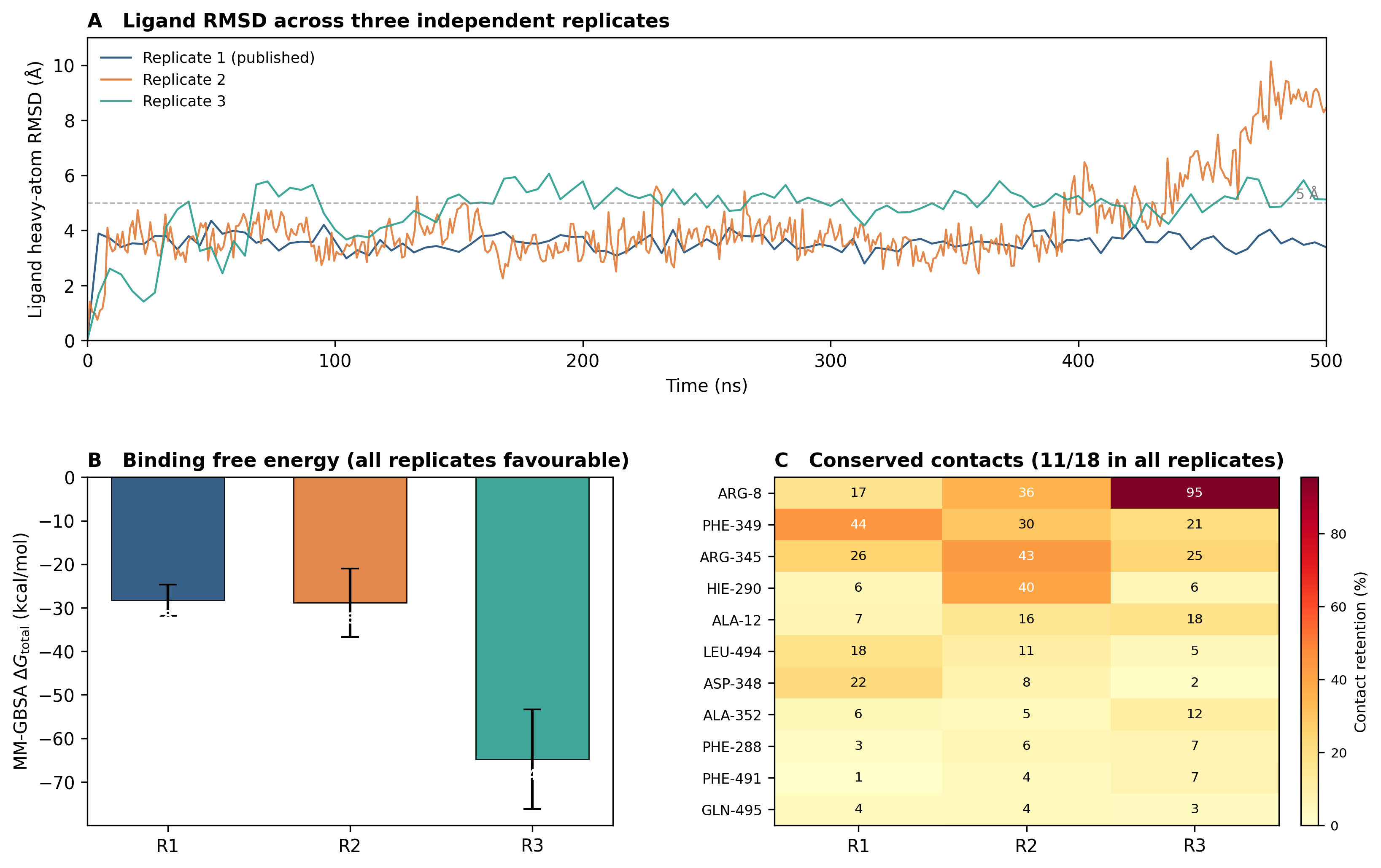

Supplement: Supplementary file 1 [file pharmaceuticals-19-00970-s001.zip › pharmaceuticals-4374763-supplementary Figure S1.png]
